# High Genetic Heterogeneity in Chinese Patients With Waardenburg Syndrome Revealed by Next-Generation Sequencing

**DOI:** 10.3389/fgene.2021.643546

**Published:** 2021-06-04

**Authors:** Sen Zhang, Hongen Xu, Yongan Tian, Danhua Liu, Xinyue Hou, Beiping Zeng, Bei Chen, Huanfei Liu, Ruijun Li, Xiaohua Li, Bin Zuo, Ryan Tang, Wenxue Tang

**Affiliations:** ^1^School of Basic Medical Sciences, Zhengzhou University, Zhengzhou, China; ^2^Precision Medicine Center, Academy of Medical Science, Zhengzhou University, Zhengzhou, China; ^3^The Second Affiliated Hospital of Zhengzhou University, Zhengzhou, China; ^4^BGI College, Zhengzhou University, Zhengzhou, China; ^5^Department of Otology, The First Affiliated Hospital of Zhengzhou University, Zhengzhou, China; ^6^Johns Hopkins University, Maryland, MD, United States; ^7^Henan Institute of Medical and Pharmaceutical Sciences, Zhengzhou University, Zhengzhou, China

**Keywords:** *PAX3*, *SOX10*, *MITF*, Waardenburg syndrome, next-generation sequencing, genetic heterogeneity

## Abstract

**Objective:**

This study aimed to explore the genetic causes of probands who were diagnosed with Waardenburg syndrome (WS) or congenital sensorineural hearing loss.

**Methods:**

A detailed physical and audiological examinations were carried out to make an accurate diagnosis of 14 patients from seven unrelated families. We performed whole-exome sequencing in probands to detect the potential genetic causes and further validated them by Sanger sequencing in the probands and their family members.

**Results:**

The genetic causes for all 14 patients with WS or congenital sensorineural hearing loss were identified. A total of seven heterozygous variants including c.1459C > T, c.123del, and c.959-409_1173+3402del of *PAX3* gene (NM_181459.4), c.198_262del and c.529_556del of *SOX10* gene (NM_006941.4), and c.731G > A and c.970dup of *MITF* gene (NM_000248.3) were found for the first time. Of these mutations, we had confirmed two (c.1459C > T and c.970dup) are *de novo* by Sanger sequencing of variants in the probands and their parents.

**Conclusion:**

We revealed a total of seven novel mutations in *PAX3*, *SOX10*, and *MITF*, which underlie the pathogenesis of WS. The clinical and genetic characterization of these families with WS elucidated high heterogeneity in Chinese patients with WS. This study expands the database of *PAX3*, *SOX10*, and *MITF* mutations and improves our understanding of the causes of WS.

## Introduction

Waardenburg syndrome (WS) is a congenital developmental disorder, which is mainly characterized by congenital sensorineural hearing loss (SNHL) and abnormal pigmentation of the iris, hair, and skin (manifests as heterochromia iridis and brilliant blue eyes, a white forelock, and premature graying, and hypopigmented skin) (Read and Newton, 1997). WS has an incidence rate of approximately 1/42,000 births and is responsible for 2–5% of cases of total congenital deafness (Read and Newton, 1997; Nayak and Isaacson, 2003). Four different types of Waardenburg syndrome (WS I∼IV) have been described based on genotypic and phenotypic variations (Read and Newton, 1997; Pingault et al., 2010). WS I is distinguished from WS II by the presence of dystopia canthorum, which is lateral displacement of the inner canthus in each eye; WS III (Klein–Waardenburg syndrome) is similar to WS I except with additional upper limb abnormalities; WS IV (Waardenburg-Shah syndrome) is characterized by general WS features as well as Hirschsprung’s disease, a disorder that causes severe blockage of the large intestine. Current research suggests that WS I and WS II are more common than WS III and WS IV (Read and Newton, 1997; Pingault et al., 2010).

Waardenburg syndrome shows a high degree of genetic heterogeneity (Hageman and Delleman, 1977; Read and Newton, 1997; Pingault et al., 2010; Song et al., 2016). Six genes have been linked to this syndrome: paired box 3 (*PAX3*) (Baldwin et al., 1992; Tassabehji et al., 1992; Hoth et al., 1993), melanocyte inducing transcription factor (*MITF*) (Tassabehji et al., 1994), SRY-box transcription factor 10 (*SOX10*) (Pingault et al., 1998; Bondurand et al., 2007), endothelin 3 (*EDN3*) (Edery et al., 1996), endothelin receptor type B (*EDNRB*) (Puffenberger et al., 1994), and snail family transcriptional repressor 2 (*SNAI2*) (Sánchez-Martín et al., 2002). *PAX3* is responsible for WS I and WS III (Baldwin et al., 1992; Tassabehji et al., 1992; Hoth et al., 1993). *SOX10*, *MITF*, and *SNAI2* are associated with WS IV (Tassabehji et al., 1994; Pingault et al., 1998; Sánchez-Martín et al., 2002; Bondurand et al., 2007). *SOX10*, *EDNRB*, and *EDN3* are found to be involved in WS IV (Puffenberger et al., 1994; Edery et al., 1996; Pingault et al., 1998; Bondurand et al., 2007). Although not currently fully understood, all these genes are involved in a complex network in neural crest cells and other derivatives (Read and Newton, 1997; Bondurand et al., 2000; Pingault et al., 2010). The interaction of these genes during the formation and development of melanocytes could be the pathogenesis of WS and other related diseases (Read and Newton, 1997; Bondurand et al., 2000; Pingault et al., 2010).

Diagnosis of WS can be difficult because all features are not present in every patient (Hageman and Delleman, 1977; Newton, 1990; Tamayo et al., 2008; Pingault et al., 2010; Yang et al., 2013). Even within a single family, patients can display different clinical manifestations due to variations in the expressivity of causative genes (Hageman and Delleman, 1977; Newton, 1990; Tamayo et al., 2008; Pingault et al., 2010; Yang et al., 2013). Therefore, genetic testing is an important method for diagnosing this disease and its subtypes (Hageman and Delleman, 1977; Read and Newton, 1997; Pingault et al., 2010; Tang et al., 2015; Song et al., 2016; Wu et al., 2016; Li et al., 2019). To date, ∼400 mutations including missense/nonsense mutations, frameshift mutations, insertions/deletions, and copy number variants (CNVs) have been identified in genes associated with WS (The Human Gene Mutation Database^1^), with most variants in genes *PAX3*, *SOX10*, and *MITF* (Chen et al., 2010; Pingault et al., 2010; Song et al., 2016). Of these variants, ∼100 mutations were identified in Chinese people. Nevertheless, there are still a number of cases unexplained at the molecular level (Pingault et al., 2010; Song et al., 2016). Discovering novel mutations will lead to a better understanding of the genetic causes of WS pathogenesis.

Recently, next-generation sequencing (NGS) has proven to be a potent tool for the identification of pathogenic mutations related to deafness, which can improve the diagnosis of genetic diseases and the detection of mutations in genes associated with different clinical manifestations (Brownstein et al., 2012; Lin et al., 2012; Tang et al., 2012; Li et al., 2019). In this study, WES was used to identify the possible pathogenic mutations of patients with SNHL or WS. A total of seven novel variants in *PAX3*, *SOX10*, and *MITF* were found, and two of them are *de novo* confirmed by Sanger sequencing of variants in the probands and their parents. Our results show that WS in China has a high degree of genetic heterogeneity and extend the mutational spectrum of WS-related genes.

## Materials and Methods

### Patients

**FIGURE 1:**
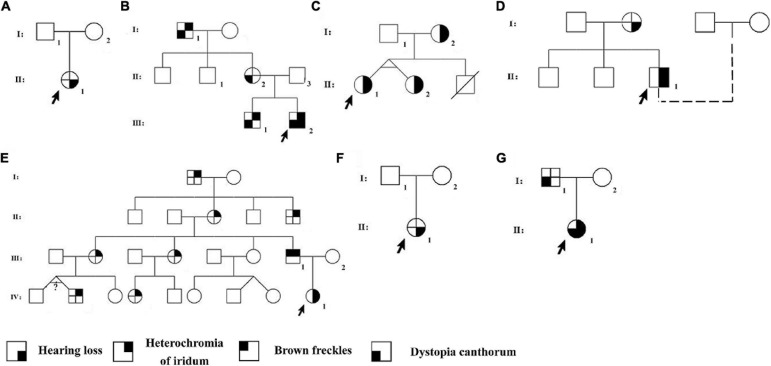
Pedigrees of the Waardenburg syndrome families. Pedigrees of families Individuals with a number assigned participated in the current study. Phenotypes of the rest of the family members were based on the relative’s description. The probands were pointed by arrows. **(A)** WS01, **(B)** WS02, **(C)** WS03, **(D)** WS04, **(E)** WS05, **(F)** WS06, and **(G)** WS07.

**FIGURE 2:**
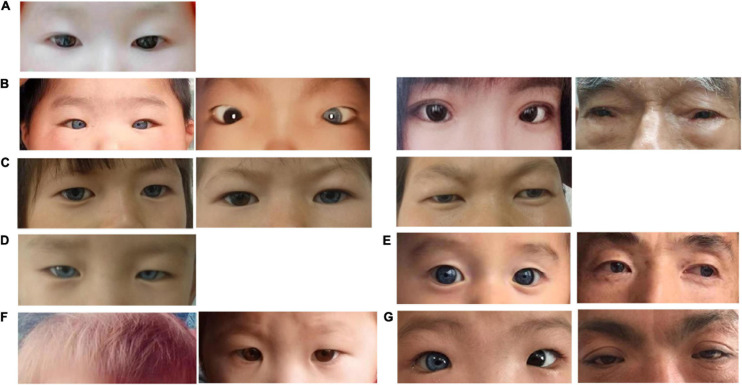
Photographs of affected individuals. **(A)** WS01-II:1 presented normal pigmentation of the iris, hair, and skin, and without dystopia canthorum. **(B)** B1, WS02-III:2; B2, WS02-III:1; B3, WS02-II:2; and B4, WS02-I:1. They all presented dystopia canthorum, while WS02-III:2 has bilateral blue iris and WS02-III:1 has unilateral. **(C)** C1, WS03-II:1; C2, WS03-II:2; and C3, WS03-I:2. They presented bilateral or unilateral blue iris. **(D)** WS04-II:1 presented complete bilateral blue iris. **(E)** E1, WS05-IV:1 presented complete bilateral blue iris. E2, WS05-III:1 presented complete bilateral blue iris and special brown freckles on the face. **(F)** F1, F2, WS06-II:1 presented yellow hair and normal iridis color. **(G)** G1, WS07-II:1 presented unilateral blue iris and dystopia canthorum. G2, WS07-II:1 presented dystopia canthorum.

From seven Han Chinese families in the Henan province, 14 patients (Table 1) and nine unaffected family members were recruited for our study and asked to perform audiological and general physical examinations (Figure 1). Furthermore, in family WS04, only WS04-II:1 was recruited because he was adopted and had lost contact with his biological family. Among the seven families, WS01 and WS06 were isolated cases, while the remaining families had multiple affected individuals (Figure 1). Photos and blood were collected after informed consent (Figure 2). This study was conducted according to the Declaration of Helsinki and approved by the institutional review board of the Medical Ethics Committee of The Second Affiliated Hospital of Zhengzhou University (Approval No. 2018008).

**TABLE 1:** Summary of clinical data for patients.

**Individual**	**Gender**	**Hearing loss**	**Blue iris**	**White forelock**	**Dystopia canthorum**	**Brown freckles**
WS01-II:1	Female	+	–	–	–	–
WS02-I:1	Male	–	+	–	+	–
WS02-II:2	Female	–	–	–	+	–
WS02-III:1	Male	–	+(Unilateral)	–	+	–
WS02-III:2	Male	+	+	–	+	–
WS03-I:2	Female	+	+	–	–	–
WS03-II:1	Female	+	+	–	–	–
WS03-II:2	Female	+	+(Unilateral)	–	–	–
WS04-II:1	Male	+	+	+	–	–
WS05-III:1	Male	–	+	–	–	+
WS05-IV:1	Female	+	+	–	–	–
WS06-II:1	Female	+	–	+	–	–
WS07-I:1	Male	–	–	–	+	–
WS07-II:1	Female	+	+(Unilateral)	–	+	–

### Clinical Investigation

All patients (medical history described by parents) received elaborate physical examinations in their hair color and skin pigmentation, joints, skeletomuscular system, digestion, ophthalmology and otology, and intelligence assessment. Patients also underwent audiological examinations, which included auditory steady-state response (ASSR), auditory brainstem response (ABR), and distortion product otoacoustic emission (DPOAE). Additionally, imageological examinations such as computerized tomography (CT) of the temporal bone and magnetic resonance imaging (MRI) were conducted. The characteristics of the patients are summarized in Table 1.

### Next-Generation Sequencing-Based Genetic Testing

In this study, WES was applied to identify the potential genetic causes for probands. A standard NGS-based genetic testing, including sample preparation and quantification, library construction, sequencing, and data analyses, was performed as previously described (Pan et al., 2020). Briefly, after library construction, the resulting libraries were hybridized to the Agilent SureSelect Human All Exon V7. Then, sequencing was carried out on an Illumina HiSeq 4000 sequencer (Illumina Inc., San Diego, CA, United States) to generate paired-end reads of 150 bp.

Data analyses were divided into bioinformatics analysis and variant interpretation. Under the framework of bcbio-nextgen^2^, we used the Burrows–Wheeler Aligner (BWA) (version 0.7.17-r1188) (Li, 2013) to align the sequencing reads to the human reference genome (GRCh37); GATK Haplotype Caller software (version 4.1.2) (McKenna et al., 2010) to identify the single nucleotide variants (SNVs) and short indels; DECoN (Fowler et al., 2016) to identify the CNVs; and Vcfanno software (version 0.3.1) (Pedersen et al., 2016) to annotate the VCF files with external database, including Clinvar (Landrum et al., 2018), ExAC (Lek et al., 2016), dbNSFP (Liu et al., 2016), 1,000 Genomes (Auton et al., 2015), and gnomAD (Karczewski et al., 2019). The filtered variants were interpreted following the guidelines of the American College of Medical Genetics and Genomics and the Association for Molecular Pathology (ACMG-AMP) (Richards et al., 2015) and the ClinGen hearing loss expert group’s recommendation on variant interpretation (Oza et al., 2018). Copy number analysis was performed from NGS data using DECoN with the bam files from the same enrichment panel and sequencing run. Paternity tests were performed on families WS01 and WS06 since the gene tests had shown mutations occurred *de novo*.

### Sanger Sequencing

**FIGURE 3:**
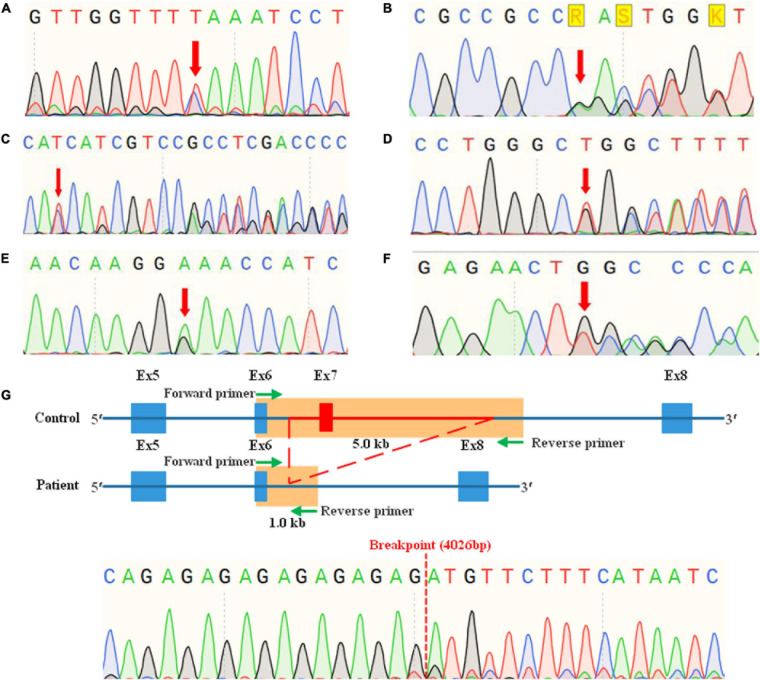
Mutation analyses of Chinese Waardenburg syndrome families WS01 to WS07 by sanger sequencing. **(A)** Heterozygous mutation c.1459C > T of *PAX3* in WS01-II:1. **(B)** Heterozygous mutation c.123del of *PAX3* in WS02-I:1, II:3, III:1, and III:2. **(C)** Heterozygous mutation c.198_262del of *SOX10* in WS03-I:2, II:1, and III:2. **(D)** Heterozygous mutation c.529_556del of *SOX10* in WS04-II:1. **(E)** Heterozygous mutation c.731G > A of *MITF* in WS05-III:3, IV:4. **(F)** Heterozygous mutation c.970dup of *MITF* in WS06-II:1. **(G)** Heterozygous mutation c.959-409_1173+3402del of *PAX3* in WS07-I:1, II:1.

Sanger sequencing was used to confirm the candidate variants detected by NGS and to conduct co-segregation analyses in family members. The specific primers (Table 2) were designed by NCBI Primer-BLAST and synthesized by Sunya Biotech Co., Ltd. (Zhengzhou, China). Conventional PCR was performed for SNVs and short indels detected in families WS01 to WS06. While long-range PCR (LR-PCR) based on nested-PCR and fragments gel-purified were performed for the CNV of patients WS07-I:1 and WS07-II:1. LR-PCR is a traditional approach to obtain CNV breakpoint junction (Woodward et al., 2005; Zhang et al., 2017), for which several primers were designed from both the proximal and the distal breakpoint regions identified, and used in different combinations until an appropriate size product was generated (Supplementary Figure 1). After PCR amplification, purification, and quality control, Sanger sequencing was run in a SeqStudio Genetic Analyzer (Thermo Scientific, United States) with a mixture of PCR products and BigDyeTM Terminator v3.1 Cycle Sequencing Kit (Applied Biosystems, Foster City, CA, United States). The sequencing results were analyzed by the SnapGene viewer (Figure 3).

**TABLE 2:** Primer pairs of the novel mutations of paired box 3 (PAX3), SRY-box transcription factor 10 (SOX10), and melanocyte inducing transcription factor (MITF).

**Mutations**	**Affected family**	**Forward primer sequence (5′–3′)**	**Reverse primer sequence (5′–3′)**	**Product length**
*PAX3*:c.1459C > T	WS01	GCCCAAACCAGTCTGGGTAAAT	GCATGACCTAAAAAGCTGCGT	471 bp
*PAX3*:c.123del	WS02	AGGACGTATGGAGCCAGTCT	GAGTCCGATGTCGAGCAGTT	351 bp
*SOX10*:c.198_262del	WS03	TGGTCTTCCAGCCCTATCCA	CAGGCGAGCTGGGCAAG	419 bp
*SOX10*:c.529_556del	WS04	CAGGGTCTCATTGCCATCCA	CAGGGCCTCACATCTTCCAA	459 bp
*MITF*:c.731G > A	WS05	GCAAACACTCGTGAATGGCA	CTGAGCAACAAATGCCGGTT	510 bp
*MITF*:c.970dup	WS06	TTCCCTTATTCCATCCACGGG	TCAGTCCCAGTTCCGAGGTT	186 bp
*PAX3*:c.959-409_1173+3402del	WS07	GAGCGCGTAATCAGTCTGGG	GGCCACATTTAGGACATGCG	19,658/15,633 bp*
	WS07	AAAATGCACAGACCCTTTCAGCA	TCTGGTTTAGCAACCGCCG	4,998/973 bp*

*The product length of the normal allele and mutated allele is separated by the slash. Reference sequence transcript: *PAX3*: NM_181459.4; *SOX10*: NM_006941.4; and *MITF*: NM_000248.3.

## Results

### Clinical Findings

A total of 14 patients from seven unrelated families were involved in this study. Before genetic testing, patients WS01-II:1 and WS06-II:1 were primarily diagnosed with SNHL, while the other 12 patients were diagnosed with WS. These diagnoses were made by otorhinolaryngologists based on the manifestation of the typical symptom of WS, such as SNHL, abnormal pigmentation, and the presence or absence of dystopia canthorum, musculoskeletal anomalies, and intestinal aganglionosis. After a genetic diagnosis, further examinations were performed on the patients WS01-II:1 and WS06-II:1. We found that the hair color of WS06-II:1 is gray, which was previously ignored, but nothing new with WS01-II:1.

Among all the patients, heterochromia iridum and deafness were the most frequent features. Ten affected individuals (10/14, 71.4%) had blue iris, of which three were heterochromia iridum; nine patients (9/14, 64.3%) had a profound sensorineural hearing impairment; six (6/14, 42.9%) had dystopia canthorum; one (1/14, 7.1%) had facial freckles; two (2/14, 14.3%) had abnormal pigmentation of hair (Figures 1, 2). Table 1 lists the clinical data of these WS patients.

Members in the same family can have different symptoms. WS03-II:1 and WS03-II:2 are identical twins; one has bilateral blue iris and the other has unilateral. According to his adoptive father, WS04-II:1 was born with a white forelock that returned to being black (we were unable to collect pictures of the patient with a white forelock). Across four generations, seven members of the family WS05 had unexpectedly brilliant blue eyes without other WS-related symptoms. However, it is impossible to confirm whether they were affected by the same mutation of the proband (WS05-IV:1) since they refused to provide blood samples.

### Molecular Etiology

Whole-exome sequencing was performed in probands of these seven families. For each sample, at least 10 Gbp raw data was generated, with more than 82% of bases having a Phred quality score *Q* ≥ 30 (Q30), 99% of the clean reads can map to the human reference genome (GRCh37/hg19), and the average sequencing depth of target regions was 100×, with 95% of target regions having coverage greater than 20× (Supplementary Table 1).

In this study, the genetic causes for all recruited patients had been confirmed, which contain a total of seven mutations in *PAX3*, *SOX10*, and *MITF*, respectively (Table 3). To our best knowledge, these mutations, including a nonsense mutation, a missense mutation, a CNV, and four frameshift mutations, have not been reported by previous studies or recorded in any public database. Further analysis by Sanger sequencing of patients and their family members shows that all the variants were present in the affected members and absent in the unaffected ones (Figure 3), and variants of probands WS01-II:1 and WS06-II:1 are *de novo*. Pathogenicity analysis of variants was according to the standards and guidelines for interpreting genetic variants proposed by the ACMG-AMP. The mutations and pathogenicity analysis were summarized in Table 3.

**TABLE 3:** Gene variants and pathogenicity analysis of patients.

**Family**	**Affected family members**	**Variants**	**Exon**	**Zygote**	**Reference**	**ACMG-AMP classification**	**ACMG-AMP criteria**
WS01	II:1	*PAX3*:c.1459C > T p.Gln487Ter Nonsense	Exon10	Heterozygous	This study	Pathogenic	PVS1, PM2, PP3, and PS2
WS02	I:1; II:2; III:1; III:2	*PAX3*:c.123del p.Gly42AlafsTer68 Frameshift	Exon2	Heterozygous	This study	Pathogenic	PVS1, PM2, PP1, PP3, and PP4
WS03	I:2; II:1; II:2	*SOX10*:c.198_262del p.Lys67AlafsTer45 Frameshift	Exon2	Heterozygous	This study	Pathogenic	PVS1, PM2, PP1, PP3, and PP4
WS04	II:1	*SOX10*:c.529_556del p.Arg177AlafsTer100 Frameshift	Exon3	Heterozygous	This study	Pathogenic	PVS1, PM2, and PP3
WS05	III:1; IV:1	*MITF*:c.731G > A p.Gly244Glu Missense	Exon8	Heterozygous	Steingrímsson et al., 1996 *	Likely Pathogenic	PS3*, PM2, PP1, and PP3
WS06	II:1	*MITF*:c.970dup p.Cys324LeufsTer36 Frameshift	Exon9	Heterozygous	This study	Pathogenic	PVS1, PM2, PS2, and PP3
WS07	I:1; II:1	c.959-409_1173+3402del Deletion	Exon7	Heterozygous	This study	Pathogenic	PVS1, PM2, and PP4

*This mutation was, for the first time, found in humans after being detected and researched by Steingrímsson et al. (1996) in mice, so the criteria “PS3” was given.

*ACMG-AMP, Association for Molecular Pathology. Reference sequence transcript: *PAX3*: NM_181459.4; *SOX10*: NM_006941.4; and *MITF*: NM_000248.3.*

### Genotype–Phenotype Correlation

The phenotypes of WS patients with *PAX3* (*n* = 7), *SOX10* (*n* = 4), and *MITF* (*n* = 3) mutations are compared in Supplementary Table 2. Among the WS patients who participated in this study, all patients with *SOX10* mutations have hearing loss, while some patients with *PAX3* (3/7) or *MITF* (2/3) have. Similarly, the symptom of the blue iris could be found in all patients with *SOX10* mutations, while it was found in some patients with *PAX3* (4/7) or *MITF* (2/3) variants. Abnormal hair pigmentation is rare in patients with *PAX3* (0/7), *SOX10* (1/4), and *MITF* (1/3) mutations. Previous reports suggested that freckles could be observed only in Chinese WS II patients with *MITF* mutations (Chen et al., 2010; Sun et al., 2016). Indeed, in this study, we found one WS II patient with *MITF* mutation has freckles. Synophridia is only present in WS I patients with *PAX3* mutations.

## Discussion

In this study, we had confirmed seven novel heterozygous variants which are the genetic causes of 14 WS patients from seven unrelated families, including c.1459C > T (nonsense), c.123del (frameshift), and c.959-409_1173+3402del (deletion) of *PAX3* (NM_181459.4), c.198_262del (frameshift) and c.529_556del (frameshift) of *SOX10* (NM_006941.4), and c.731G > A (missense) and c.970dup (frameshift) of *MITF* (NM_000248.3) (Table 3). Among 14 patients, seven each were classified as WS I and WS II, respectively, based on their phenotypes and genotypes, showing that WS I and WS II were two major WS subtypes (Read and Newton, 1997; Pingault et al., 2010). While mutations in *PAX3* were the major causes for WS I (7/7), *SOX10* (4/7), and *MITF* (3/7) were two major causative genes attributable to WS II. Our findings had extended the mutational spectrum of WS-related genes and revealed high genetic heterogeneity in Chinese WS patients (Yang et al., 2013; Sun et al., 2016; Liu et al., 2020).

To explore the genotype–phenotype correlation, we compared the phenotypes between WS patients with *PAX3*, *SOX10*, and *MITF* mutations (Supplementary Table 2). Several reports had shown that the clinical features of WS II caused by *SOX10* and *MITF* mutations were indistinguishable, except that freckle was frequent in WS II probands with *MITF* mutation (Chen et al., 2010; Toriello, 2011; Sun et al., 2016). Indeed, in this study, freckle seems to be unique for patients with *MITF* mutations (1/3) but was absent in those with *PAX3* (0/7) or *SOX10* (0/4) mutations. Dystopia canthorum is a rebarbative but crucial clinical feature, because of its value in distinguishing WS I and WS II, but it is not completely applicable for Chinese WS I patients (Sun et al., 2016; Morimoto et al., 2018; Suzuki et al., 2018; Minami et al., 2019). Herein, we had an interesting finding that the synophridia, even though a minor symptom, was only present in WS patients (5/7) but absent in WS II patients (0/7). Our results may have shown the clinical differences between WS II patients with *SOX10* and *MITF* mutations, and between WS II and WS I. However, gene test is as necessary as clinical investigation for the accurate diagnosis and subtype confirmation (Hageman and Delleman, 1977; Read and Newton, 1997; Pingault et al., 2010; Song et al., 2016; Li et al., 2019).

Mutations in *PAX3*, *SOX10*, and *MITF* were the most common genetic causes for WS and responsible for almost all Chinese WS patients (Chen et al., 2010; Wu et al., 2016; Liu et al., 2020). Beyond that, to date, several WS cases associated with mutations in *ENDRB*, *EDN3*, and *SNAI2* had been reported (Sánchez-Martín et al., 2002; Pingault et al., 2010; Xiong et al., 2015; Somashekar et al., 2019), but the situation is a bit different in Chinese. There were two reported cases of WS type I caused by mutations in the *EDNRB* gene (Cheng et al., 2019; Li et al., 2019), which is different from the cases in other races (WS II or IV) (Pingault et al., 2010; Issa et al., 2017). Of the few reports about WS type II being caused by mutations in the *EDN3* gene, there was no Chinese case reported. Only one research group reported *SNAI2* mutations caused WS within two unrelated WS II patients (Sánchez-Martín et al., 2002), which had been questioned recently (Song et al., 2016; Mirhadi et al., 2020). To understand the differences of WS among different races, we need further research on the pathogenesis of WS and more accurate diagnostic means.

The deficiency of melanocytes, the neural crest (NC) derivatives, is common to various WS types (Bondurand et al., 2000; Pingault et al., 2010; Song et al., 2016), which is responsible for the phenotypes of pigmentation defects and hearing loss (Steel and Barkway, 1989). *PAX3* encodes a DNA-binding transcription factor, consisting of a paired box (PD) encoded by exons 2, 3, and 4, the homeodomain (HD) by exons 5 and 6, C-terminal transcriptional activation domain by exons 7 and 8 (Read and Newton, 1997; Wildhardt et al., 2013). It is indispensable in the development of somites, skeletal muscle, and the neural crest cells (NCC) and their derivatives like melanocytes. It can cooperate with *SOX10* to regulate the expression of the *MITF* promoter (Pingault et al., 2010). *PAX3*:c.123del and c.959-409_1173+3402del mutations are predicted to activate the nonsense-mediated mRNA decay (NMD) machinery (Khajavi et al., 2006), thereby resulting in haploinsufficiency, which might be the disease-causing mechanism for WS I. *PAX3*:c.1459C > T mutation is located in the exon 10 and could only influence the isoform *PAX3e* (Wang, 2006), which most likely pathogenic mechanism is haploinsufficiency (Barber et al., 1999).

SRY-box transcription factor 10 encodes a transcription factor that contains an HMG (high mobility group) DNA binding domain and a C-terminal transactivation domain (Chan et al., 2003). In the early development of NC, *SOX10* plays an important role in promoting cell survival and maintaining the multipotency of NC stem cells (Kapur, 1999; Kelsh, 2006; Pingault et al., 2010; Stolt and Wegner, 2010). Besides synergy with *PAX3* to regulate the expression of *MITF*, it also can directly regulate the expression of genes important for melanin synthesis, suggesting the importance for melanocyte differentiation (Bondurand et al., 2000; Lee et al., 2000; Potterf et al., 2000; Verastegui et al., 2000; Jiao et al., 2004; Wegner, 2005; Pingault et al., 2010). It is also crucial for the peripheral nervous system like sensory, sympathetic, and enteric ganglia and along nerves (Bondurand et al., 2000; Pingault et al., 2010). *SOX10*: c.198_262del and c.529_556del are located in the HMG domain and predicted to activate the NMD machinery, resulting in haploinsufficiency.

Melanocyte inducing transcription factor, a basic helix–loop–helix leucine zipper (bHLHZip) protein, is the key transcription factor of melanocyte development. The bHLHZip structure binds DNA by basic domain, dimerizes through HLH domain, and is stabilized via the Zip domain (Hodgkinson et al., 1993; Steingrímsson et al., 1994). The C-terminal of MITF contributes to defining the target genes by a serine-rich transcriptional activation domain. Mice with *MITF* mutations show reduced or absent pigmentation, deafness, and small or absent eyes, etc. (Yasumoto et al., 1995; Bertolotto et al., 1998; Steingrímsson et al., 2004; Pingault et al., 2010). *MITF*:c.970dup is a frameshift mutation and predicted to activate the NMD machinery, leading to haploinsufficiency. *MITF*:c.731G > A (p.Gly244Glu) is the genetic cause of III:1 and IV:1 of family WS05 and might be responsible for the other seven affected individuals (Figure 1). We had noticed that only one had hearing loss and blue iris while the other eight only had blue iris in this family, although Song et al. (2016) had suggested that nearly 90% of patients with *MITF* have hearing loss. The Gly244Glu mutation of MITF was found in humans for the first time, while the mouse model with the same mutation (MITF^*Mi–b*^) had been found by Steingrímsson et al. (1996). The phenotype of *MITF*^*Mi–b*^ homozygous animals is mild compared with loss-of-function *mi* alleles. Gly244 would lie at the very beginning of the second helix, close to the protein-DNA interface. The Gly244Glu alteration is at the junction of the loop and helix 2 of the protein. The MITF^*Mi–b*^ protein largely spares dimerization function, while it is defective in its ability to bind DNA. However, the DNA binding function can be partially compensated by a wild-type partner in the dimer, since MITF^*Mi–b*^ is capable of forming TFE3 (Transcription factor E3) heterodimeric complexes which had a stronger DNA binding than the MITF^*Mi–b*^ homodimers. It may explain why the mutation c.731G > A resulting in a less-severe phenotype in the family WS05.

WS has high genetic heterogeneity and highly variable phenotype expressivity (Hageman and Delleman, 1977; Newton, 1990; Read and Newton, 1997; Tamayo et al., 2008; Pingault et al., 2010; Yang et al., 2013; Song et al., 2016), which makes the diagnosis challenging. NGS of numerous genes is allowed in a single test with lower turnaround time, cost, and higher throughput, which makes it ideal for figuring out the exact genetic mechanism (Brownstein et al., 2012; Tang et al., 2012, 2015). Mutations in *PAX3* are responsible for WS I and WS III in most cases; however, using WES, we had detected a heterozygous nonsense mutation of *PAX3*:c.1459C > T in an SNHL patient (WS01-II:1). To our best knowledge, this is the first mutation found in exon 10 of *PAX3* and results in a premature stop codon, which is very close to the normal ending (487/506) (Boudjadi et al., 2018). We suspect that this is why SNHL is the only symptom of WS01-II:1, even though previous studies argued that there was no correlation between genotype and phenotype of WS caused by *PAX3* mutations (Tassabehji et al., 1995; Boudjadi et al., 2018). Patient WS06-II:1 was also diagnosed with SNHL initially, before being corrected to WS type II after the genetic testing in which a heterozygous *de novo* mutation *MITF*: c.970dup was detected. Besides, the other five families with classic symptoms and clear family histories, these two cases in particular highlight the superiority of NGS in the diagnosis of WS.

In conclusion, the clinical and genetic characteristics of one SNHL patient and six Chinese families of WS had been investigated in this study. Altogether, seven novel pathogenic/likely pathogenic variants in the *PAX3*, *SOX10*, and *MITF* were identified. Our results support that NGS is a useful diagnostic procedure for the diagnosis and subtype differentiation of WS. This report reveals the highly genetic heterogeneity and variable phenotype in Chinese patients with WS and will contribute to a better understanding of the WS by extending the mutational spectrum of WS-related genes.

## Data Availability Statement

According to national legislation/guidelines, specifically the Administrative Regulations of the People’s Republic of China on Human Genetic Resources (http://www.gov.cn/zhengce/content/2019-06/10/content_5398829.htm, http://english.www.gov.cn/policies/latest_releases/2019/06/10/content_281476708945462.htm), no additional raw data is available at this time. Data of this project can be accessed after an approval application to the Bio-Med Big Data Center, NODE. Please refer to https://www.biosino.org/node/project/detail/OEP001401 for detailed application guidance. The accession code OEP001401 should be included in the application.

## Ethics Statement

The studies involving human participants were reviewed and approved by the institutional review board of the Medical Ethics Committee of The Second Affiliated Hospital of Zhengzhou University. Written informed consent to participate in this study was provided by the participants’ legal guardian/next of kin.

## Author Contributions

WT and HX: study design. BC, HL, RL, XL, and BZu: patient phenotypic analysis and genetic counseling. SZ, YT, XH, BZe, HL, and RL: next−generation sequencing and Sanger sequencing. HX, DL, SZ, and YT: data analysis and variant interpretation. SZ, HX, DL, WT, and RT: writing and review of original draft of the manuscript. RT: language editing of original draft of the manuscript. All authors have read and approved the final manuscript.

## Conflict of Interest

The authors declare that the research was conducted in the absence of any commercial or financial relationships that could be construed as a potential conflict of interest.

## Footnotes

1 www.hgmd.cf.ac.uk/

2 https://github.com/bcbio/bcbio-nextgen

## Supplementary Material

Supplementary Figure 1 LR-PCR for the identification of the large deletion, *PAX3*:c.959-409_1173+3402del.

Supplementary Table 1 Detailed information for quality control of WES data.

Supplementary Table 2 Phenotypes in patients with *PAX3*, *SOX10*, and *MITF* mutations.
