# Supplementary figures and images for: High Genetic Heterogeneity in Chinese Patients With Waardenburg Syndrome Revealed by Next-Generation Sequencing
Source: Front Genet. 2021 Jun 4;12:643546. doi: 10.3389/fgene.2021.643546 (PMC8212959; doi:10.3389/fgene.2021.643546)

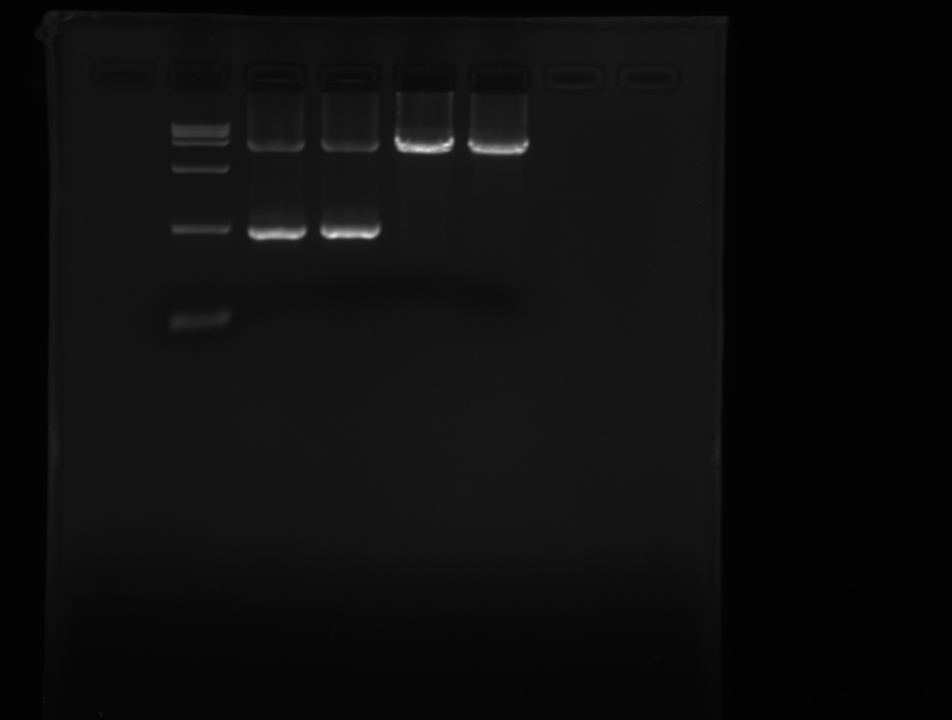

Supplement: Supplementary Figure 1 — LR-PCR for the identification of the large deletion, PAX3:c.959-409_1173+3402del. [file Data_Sheet_1.zip › Figure S1 RAW.Tif]

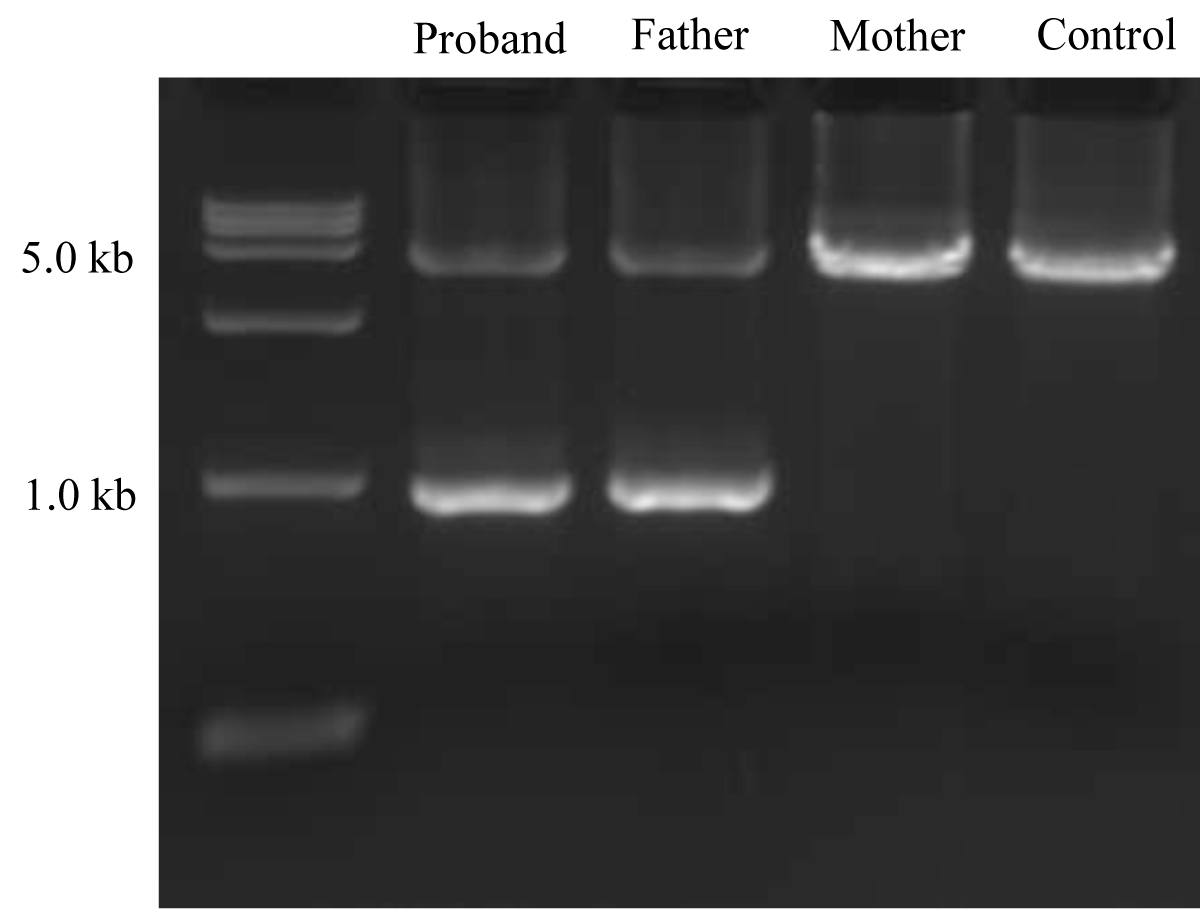

Supplement: Supplementary Figure 1 — LR-PCR for the identification of the large deletion, PAX3:c.959-409_1173+3402del. [file Data_Sheet_1.zip › Figure S1.tif]
