# Supplementary material for: High Genetic Heterogeneity in Chinese Patients With Waardenburg Syndrome Revealed by Next-Generation Sequencing
Source: Front Genet. 2021 Jun 4;12:643546. doi: 10.3389/fgene.2021.643546 (PMC8212959; doi:10.3389/fgene.2021.643546)
Supplement: Supplementary Figure 1 — LR-PCR for the identification of the large deletion, PAX3:c.959-409_1173+3402del. [file Data_Sheet_1.zip › Table S1.doc]

## Table S1 Detailed information for quality control of WES data.

| **Family** | **Sample** | **Datasize(G)** | **Q30** | **Mapping_rate** | **Avg^a^** | **Cov1X^b^** | **Cov20X^c^** |
| --- | --- | --- | --- | --- | --- | --- | --- |
| **WS01** | WS01-Ⅰ:1 | 11.79 | 0.945 | 1 | 105.4 | 0.996 | 0.971 |
| **WS02** | WS02-Ⅲ:2 | 13.25 | 0.948 | 0.999 | 102.6 | 0.989 | 0.954 |
| **WS03** | WS03-Ⅱ:1 | 11.84 | 0.828 | 0.997 | 126.3 | 0.998 | 0.983 |
| **WS04** | WS04-Ⅱ:1 | 18.9 | 0.919 | 0.997 | 150.9 | 0.999 | 0.987 |
| **WS05** | WS05-Ⅳ:1 | 12.73 | 0.946 | 0.999 | 102.4 | 0.996 | 0.957 |
| **WS06** | WS06-Ⅱ:1 | 11.90 | 0.951 | 0.999 | 106.6 | 0.996 | 0.970 |
| **WS07** | WS07-Ⅱ:1 | 10.93 | 0.877 | 0.999 | 114.3 | 0.995 | 0.962 |

a: Avg, the average sequencing depth of targeted regions; b, c: Cov1X or Cov20X, means the percentage of target regions had coverage greater than 1X or 20X, range 0 to 1.
