# Supplementary material for: High Genetic Heterogeneity in Chinese Patients With Waardenburg Syndrome Revealed by Next-Generation Sequencing
Source: Front Genet. 2021 Jun 4;12:643546. doi: 10.3389/fgene.2021.643546 (PMC8212959; doi:10.3389/fgene.2021.643546)
Supplement: Supplementary Figure 1 — LR-PCR for the identification of the large deletion, PAX3:c.959-409_1173+3402del. [file Data_Sheet_1.zip › Table S2.doc]

## Table S2 Phenotypes in patients with *PAX3*, *SOX10* and *MITF* mutations.

| **Phenotypes** | **PAX3** | **SOX10** | **MITF** |
| --- | --- | --- | --- |
| **Hearing loss** | **3** | **4** | **2** |
| **Blue iris** | **4** | **4** | **2** |
| **Abnormal hair pigmentation** | **0** | **1** | **1** |
| **Freckles** | **0** | **0** | **1** |
| **Synophridia** | **5** | **0** | **0** |
| **Total** | **7** | **4** | **3** |
